# In Memoriam: Professor Marcello Marcondes

**DOI:** 10.1590/2175-8239-JBN-2024-IM002en

**Published:** 2024-11-08

**Authors:** Vanda Jorgetti, Roberto Zatz

**Affiliations:** 1 Universidade de São Paulo, Faculdade de Medicina, São Paulo, SP, Brazil.

**Figure 1:**
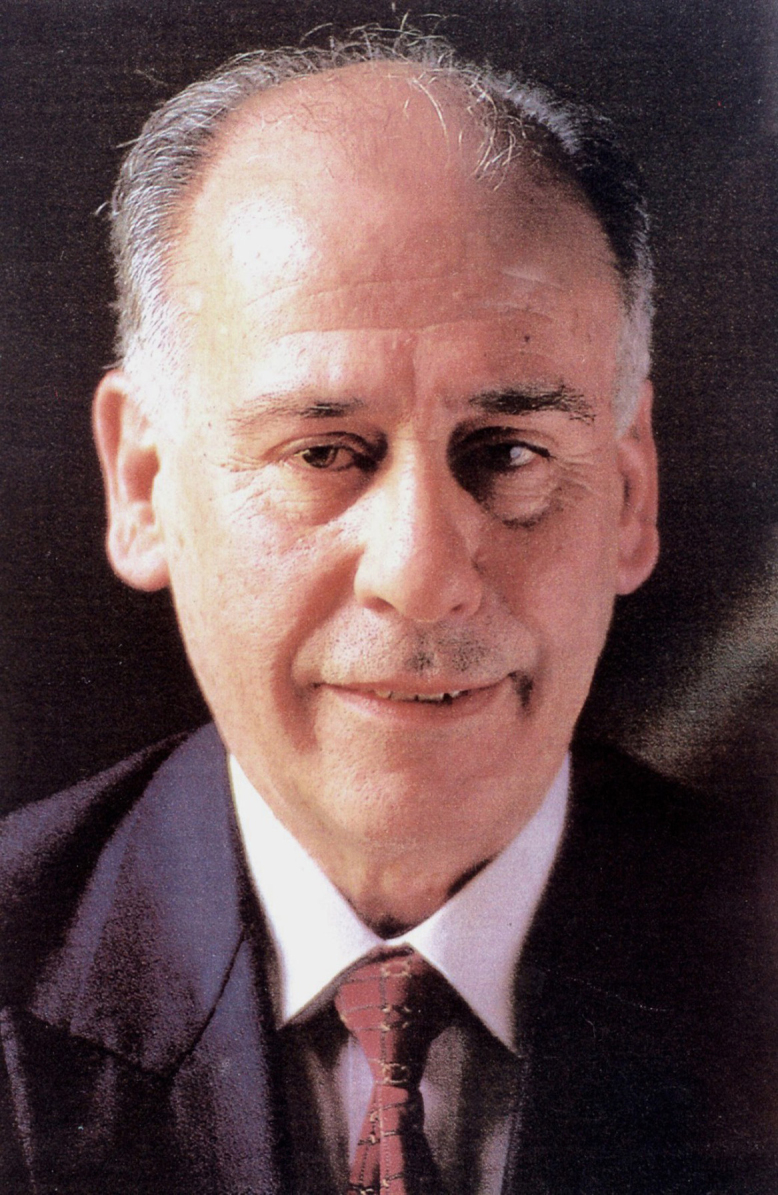
Professor Marcello Marcondes.

Professor Marcello Marcondes Machado (Figure 1) was born in São Paulo in 1933. He graduated in Medicine from the Medical School at the *Universidade de São Paulo* in 1958 and obtained his Doctorate in 1961.

Between 1962 and 1964, Prof. Marcondes was a Research Fellow at Washington University in St. Louis, United States. He was part of the group headed by Prof. Neal S. Bricker, a prominent figure in global nephrology, and contributed to major research projects on chronic kidney disease, the results of which laid the foundations for our current understanding of this condition^1,2^.

In 1968, Prof. Marcondes actively participated in the renovations of the *Universidade de São Paulo*. In that same year, he was one of the creators of the Experimental Medicine Course, which left a lasting legacy in the history of medical education. In the years that followed, he established a pioneering group of young physicians dedicated simultaneously to teaching, research, and patient care. From this group emerged several professionals who, inspired by his example, and constantly encouraged and supported by him, developed post-doctoral projects at prestigious institutions abroad. These physicians were welcomed back by him upon their return, and later became prominent in their roles as nephrologists and educators.

Professor Marcondes was one of the founders of the University Hospital, whose significance for the population care and the professional training of countless FMUSP graduates is beyond comment. Around the same time, he established the Renal Physiopathology Laboratory (LIM 16), which remains extremely active to this day, bringing together numerous healthcare professionals, both medical and non-medical, to investigate the mechanisms underlying chronic kidney diseases and their complications.

In 1977, Prof. Marcondes was one of the leading developers of the Nephrology Division at FMUSP, serving as its chair from 1978 to 1985, and as a full professor from 1985 to 2003, lending it the features it retains to this day. Upon his retirement, one of the most remarkable aspects of his administration became evident: eight associate professors - thus highly qualified academic professionals - competed for the position of full professor that he was stepping down from.

Professor Marcondes did not confine himself to Nephrology. He made an even greater contribution as the Director of FMUSP, a position he held from 1994 to 1998. His administration was characterized by the same firm support for teaching and research that he consistently provided as head of Nephrology.

He was one of the great architects of the strength of Nephrology at FMUSP, fostering an environment of harmony, cooperation and staff acquisition. Professor Marcondes was indeed one of the giants of Brazilian Nephrology, but his contributions transcended the boundaries of a single discipline. His legacy and memory will live on for generations to come.
