# Supplementary material for: In Memoriam: Professor Marcello Marcondes
Source: J Bras Nefrol. 2024 Nov 8;46(4):e2024IM002. doi: 10.1590/2175-8239-JBN-2024-IM002en (PMC11548864; doi:10.1590/2175-8239-JBN-2024-IM002en)
Supplement: Translation

# Translation: In Memoriam: Prof. Marcello Marcondes

**DOI:** 10.1590/2175-8239-JBN-2024-IM002pt

**Authors:** Vanda Jorgetti, Roberto Zatz

**Affiliations:** 1 Universidade de São Paulo, Faculdade de Medicina, São Paulo, SP, Brasil.

O Prof. Marcello Marcondes Machado (Figura 1) nasceu em São Paulo, em 1933. Formou-se em Medicina pela Faculdade de Medicina da Universidade de São Paulo em 1958 e obteve o título de Doutor em Medicina em 1961.

**Figura 1:** Prof. Marcello Marcondes.

Entre 1962 e 1964, o Prof. Marcondes foi Fellow de Pesquisa na Washington University, em St. Louis, Estados Unidos. Participou do grupo chefiado pelo Prof. Neal S. Bricker, uma figura maiúscula da Nefrologia mundial, e colaborou em importantes pesquisas sobre doença renal crônica, cujos resultados formaram a base da compreensão que temos hoje sobre essa condição^1,2^.

Em 1968, o Prof. Marcondes participou ativamente da reforma da Universidade de São Paulo. Naquele mesmo ano, foi um dos criadores do Curso Experimental de Medicina, que deixou um legado duradouro na história do ensino médico. Nos anos subsequentes, construiu um pioneiro grupo de jovens docentes dedicados simultaneamente ao ensino, à pesquisa e à assistência. Desse grupo surgiram vários quadros que, inspirados em seu exemplo, e sempre estimulados e apoiados por ele, desenvolveram projetos de pós-doutorado em instituições de prestígio no exterior. Esses médicos foram acolhidos por ele ao retornar e posteriormente tiveram destaque em sua atuação como nefrologistas e como educadores.

O Prof. Marcondes foi um dos fundadores do Hospital Universitário, cuja importância para a assistência à população e para a formação profissional de incontáveis egressos da FMUSP dispensa comentários. Na mesma época, criou o Laboratório de Fisiopatologia Renal (LIM 16), até hoje extremamente ativo, agregando inúmeros profissionais de saúde, médicos ou não, na pesquisa dos mecanismos das doenças renais crônicas e de suas complicações.

Em 1977, o Prof. Marcondes foi um dos principais criadores da disciplina de Nefrologia da FMUSP, da qual foi regente entre 1978 e 1985, e professor titular de 1985 a 2003, imprimindo-lhe as feições que conserva até hoje. Ao aposentar-se, tornou-se evidente um dos aspectos mais marcantes de sua gestão, o de formador de quadros: oito professores associados – portanto, quadros acadêmicos altamente qualificados – concorreram ao cargo de professor titular que ele deixava.

O Prof. Marcondes não se limitou à Nefrologia. Contribuiu de forma ainda mais ampla na qualidade de diretor da FMUSP, cargo que exerceu entre 1994 e 1998. Sua gestão foi caracterizada pelo mesmo firme apoio ao ensino e à pesquisa que sempre prestou como chefe de disciplina.

O Prof. Marcondes foi um dos grandes artífices da pujança da Nefrologia da FMUSP, sempre em ambiente de harmonia, cooperação e agregação de quadros. Foi um dos gigantes da Nefrologia brasileira, sim, mas sua contribuição transcendeu os limites de uma única disciplina. Seu legado e sua lembrança permanecerão por muitas gerações.
